# Probiotic-Derived Exopolysaccharides Enhance Mesenchymal Stem Cell-Mediated Fibroblast Repair under Hyperglycemic Conditions

**DOI:** 10.1021/acsomega.6c07182

**Published:** 2026-09-29

**Authors:** Mesude Bicer, Mert Akdur, Esra Battal, Fatma Sener, Irem Yilmaz, Özkan Fidan

**Affiliations:** † Department of Bioengineering, Faculty of Life and Natural Sciences, Abdullah Gul University, Kayseri 38080, Turkiye; ‡ Department of Bioengineering, Graduate School of Abdullah Gul University, Abdullah Gul University, Kayseri 38080, Turkiye

## Abstract

Diabetic wounds are closely associated with persistent oxidative stress, decreased fibroblast activity, and delayed tissue regeneration under hyperglycemic conditions. These pathological features particularly complicate wound healing and often lead to chronic non-healing wounds. Recently, mesenchymal stem cells (MSCs) and probiotic-derived biomolecules have attracted attention as potential agents to improve tissue repair. In this study, we examined whether the combination of adipose-derived mesenchymal stem cell-conditioned medium (ADMSC-CM) and bacterial exopolysaccharides (EPSs) could improve fibroblast responses under diabetes-like conditions. EPSs were extracted from *Lactobacillus plantarum* and *Lactobacillus brevis*, purified and preliminarily characterized prior to subsequent cell-based experiments. To stimulate a diabetic microenvironment, L929 fibroblasts were exposed to high-glucose conditions prior to treatment with ADMSC-CM, purified EPSs, or their combination. Cell viability, oxidative stress levels, apoptosis, and migration potential were assessed using the MTT assay, DCFH-DA staining, acridine orange/propidium iodide staining, and scratch assay, respectively. Interestingly, the combination of ADMSC-CM with *L. brevis*-derived EPS showed the strongest effect. This treatment improved fibroblast viability and wound closure under hyperglycemic conditions while significantly reducing intracellular ROS levels and apoptotic cell death. EPS derived from *L. plantarum* also showed supportive effects, but these regenerative responses were relatively weaker. Both EPS fractions were found to be biocompatible at 1000 μg/mL. Overall, these findings suggest that probiotic-derived EPS can enhance ADMSC-mediated fibroblast repair in a hyperglycemic microenvironment and serve as a supportive bioactive agent in diabetic wound healing.

## Graphical abstract

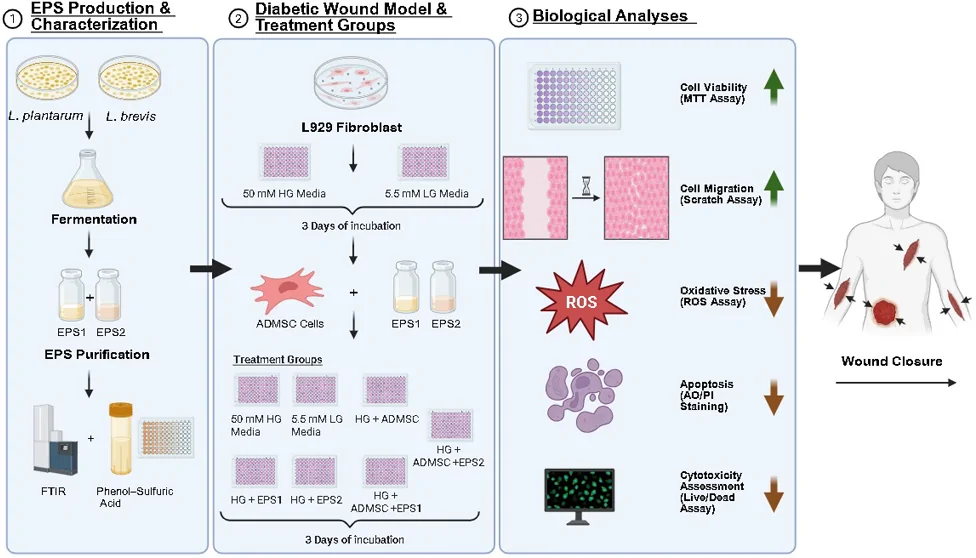

## 1 Introduction

Diabetic wounds, most notably diabetic foot ulcers, represent severe chronic complications of diabetes due to their frequent recurrence and delayed healing. One of the underlying factors is persistent hyperglycemia, leading to vascular and neural dysfunction. Under these conditions, compromised skin barrier integrity leads to hypoxia, impaired angiogenesis, chronic inflammation and an elevated risk of infection.^1^ Lifetime incidence of foot ulcers among diabetic patients ranges from 15% to 25%, with recurrence rates hitting 40% within the first year and more than 50% within five years.^2^ This condition frequently progresses to lower extremity amputation and leads to a substantial decrease in quality of life.^2^ Several therapeutic approaches such as hyperbaric oxygen therapy, growth factors, negative pressure wound therapy and biomaterial-based wound dressings are currently employed in clinical settings.^3^ Despite these therapeutic advancements, their efficacy remains limited because of high costs, long-term treatment and the need for complex clinical management.^1,2^

Wound healing is a dynamically-regulated biological process encompassing four overlapping phases: hemostasis, inflammation, proliferation and remodeling. Proper tissue repair requires fibroblasts to function in coordination with extracellular matrix deposition, angiogenesis and re-epithelialization.^2,4^ Under diabetic conditions, this coordinated process is seriously disrupted. The excessive accumulation of reactive oxygen species (ROS), sustained inflammatory signaling and biofilm formation impair tissue repair and lead to chronic wounds.^1^ These pathological features highlight the limited capacity of endogenous repair mechanisms in diabetes and draw attention to the need for alternative regenerative approaches that can restore cellular function within the diabetic microenvironment.

ADMSCs are considered promising candidates in regenerative medicine due to their accessibility, paracrine signalling activities and proliferative capacity.^5^ These stem cells facilitate tissue repair through immunomodulatory activity, specifically promoting the polarization of macrophages from the pro-inflammatory M1 phenotype to the anti-inflammatory M2 phenotype. Additionally, they secrete important trophic factors, including VEGF, FGF, HGF, and IGF-1, which support angiogenesis and tissue regeneration.^6,7^ Despite their therapeutic properties, the diabetic microenvironment, characterized by hypoxia, elevated protease activity and microbial infection, negatively impacts the survival of MSC and therapeutic efficacy.^8,9^ Consequently, there is growing interest in approaches based on bioactive compounds that enhance MSC function under hyperglycemic conditions.

Exopolysaccharides (EPS) derived from probiotic lactic acid bacteria (LAB) represent promising bioactive macromolecules with potential therapeutic applications. These polymers are high-molecular-weight carbohydrates secreted by bacteria, particularly under environmental stress.^10^ LAB strains are widely utilized for EPS production due to their biocompatibility, easy of cultivation and favorable safety profile.^11,12^ During fermentation, these bacteria produce EPS as part of their adaptive response to environmental fluctuations, including changes in pH, osmotic pressure and temperature.^13^ Beyond their structural roles, LAB-derived EPSs exhibit potent antioxidant, anti-inflammatory, antimicrobial and immunomodulatory properties.^14–16^ Furthermore, EPSs have been shown to accelerate fibroblast migration, increase collagen deposition and regulate signaling pathways such as TGF-β1/SMAD associated with angiogenesis and tissue repair.^17^ Among different LAB species, EPSs obtained from *Lactobacillus plantarum* and *Lactobacillus brevis* have been used in the treatment of diabetic wounds. These polysaccharides have been shown to be associated with improving fibroblast capability, increasing cell migration and modulating inflammatory responses in the wound microenvironment.^18–20^ EPS obtained from *L. plantarum* has been shown to suppress inflammatory signaling pathways and increase cell migration, while *L. brevis* has been shown to be associated with improving immune regulation, reducing microbial load and increasing granulation tissue formation.^21,22^ It has also been shown EPS produced by the *L. plantarum* EI6 strain is not toxic to human dermal fibroblast (HDF) cells and promotes the proliferation and migration of keratinocytes, fibroblasts and human splenic fibroblast (HSF) cells.^23^ These findings demonstrate that EPSs produced by LAB, particularly *L. plantarum* and *L. brevis*, have significant potential for wound healing and tissue regeneration.

Although the individual therapeutic roles of ADMSCs and probiotic-derived EPS have been reported, their combined effects on diabetic wound healing remain poorly understood. Therefore, this study investigates the effects of EPS derived from *L. plantarum* and *L. brevis* in combination with ADMSC-CM in a stimulated hyperglycemic wound environment. These combined effects were evaluated by cell viability, migration, apoptosis and oxidative stress. Ultimately, this study offers a novel perspective on integrating probiotic-derived polysaccharides with stem cell-mediated repair mechanisms to treat diabetic wounds.

## 2 Materials and Methods

### 2.1 Culture of Microorganisms and Stem Cells

Among *Lactobacillus* strains, *L. plantarum* DSM 20174 and *L. brevis* DSM 20054, were chosen for EPS extraction. Seed cultures were prepared by inoculating the strains into De Man, Rogosa and Sharpe broth (MRS broth, Merck) and incubated at 28 °C for 48h with agitation at 220 rpm to obtain seed cultures. Human ADMSCs were kindly provided by S.S. Hakki (Selcuk University) and were originally isolated from human palatal adipose tissue. These cells had been previously characterized according to the criteria of the International Society for Cellular Therapy as described in previous studies.^24,25^ ADMSCs were used without further modification, expanded under standard conditions and maintained at 37 °C in a humidified atmosphere containing 5% CO_2_ using a BINDER APT.line C150 incubator (BINDER GmbH, Germany). Growth media consisted of Dulbecco’s Modified Eagle Medium (DMEM; Sigma-Aldrich (Gillingham, U.K.)) supplemented with 10% heat-inactivated fetal bovine serum (FBS; Gibco, Thermo Fisher Scientific, Waltham, MA, USA), 2 mM l-glutamine, 100 U/mL penicillin, 100 μg/mL streptomycin, and 5 ng/mL basic Fibroblast Growth Factor (bFGF; Peprotech, U.K.). Unless otherwise specified, all supplements were obtained from Sigma-Aldrich (Gillingham, U.K.). The culture medium was replenished every 2–3 days, and cells were passaged upon reaching 70–80% confluence. For all downstream experiments, cells between passages 7 and 11 were used.^26^

### 2.2 Production and Characterization of Bacterial Exopolysaccharides (EPS)

#### 2.2.1 Shake Flask Cultivation and EPS Extraction

EPS extraction followed the protocol of Wang et al. (2023) with minor modifications. Shake flask cultures (3 × 150 mL for each strain) were inoculated with 1% (v/v) of the respective seed culture and incubated at 28 °C with 220 rpm agitation for 48 h. Following incubation, the cultures were heat-treated at 100 °C for 10 min to inactivate enzymes that might degrade the accumulated EPSs and eliminate potential contaminants. The cultures were then centrifuged at 13,523*g* for 20 min at 4 °C. To precipitate and eliminate remaining proteins, the supernatants were treated with 5% (w/v) trichloroacetic acid (or acetic acid) at 4 °C for 5 h, followed by a second centrifugation under the same parameters to pellet the denatured proteins. The clarified supernatants were mixed with chilled absolute ethanol at a 1:1 (v/v) ratio and incubated overnight at 4 °C to precipitate the crude EPS. The precipitates were recovered via centrifugation and dissolved in ultrapure water. For purification, the dissolved EPS solutions were dialyzed using a 14 kDa molecular weight cut-off (MWCO) membrane (Bio Basic) against distilled water at 4 °C for 24 h; the dialysis medium was stirred every 2 h and replaced once. Finally, the purified EPS solutions were lyophilized to obtain a dry powder and stored at −20 °C for subsequent analysis.^27^

#### 2.2.2 EPS Quantification and Characterization

Total carbohydrate content of the extracted EPSs was quantified using the phenol-sulfuric acid method with glucose as a standard.^28^ Working standard solutions (0–400 μg/mL) were prepared from a 1 mg/mL glucose stock solution and lyophilized EPS samples were dissolved in distilled water to a final concentration of 1 mg/mL.

Briefly, 500 μL of each standard or sample solution was mixed with 500 μL of a chilled 5% (w/v) phenol solution in glass tubes, followed by the rapid addition of 2.5 mL of concentrated sulfuric acid. The mixtures were incubated at room temperature for 20 min. Subsequently, 200 μL of each reaction mixture was transferred to a 96-well microplate, and the absorbance was measured at 490 nm using an iMarkTM Microplate Absorbance Reader (Bio-Rad Laboratories, Hercules, CA, USA). All measurements were performed in triplicate, and carbohydrate concentrations were calculated using the resulting glucose standard curve.^29^

For structural characterization, Fourier-transform infrared (FT-IR) spectra of the *L. plantarum* and *L. brevis* EPS samples were recorded using Nicolet 6700 FT-IR spectrometer equipped with an Attenuated Total Reflectance (ATR) accessory (Thermo Fisher Scientific, Waltham, MA, USA). Spectra were acquired across the wavenumber range of 400–4000 cm^–1^ to identify the functional groups present in the EPS.

### 2.3 Culture of L929 Fibroblasts and Establishment of the Hyperglycemic Model

L929 murine fibroblast cells (ATCC CCL-1) were kindly provided by a collaborating laboratory (Assist. Prof. Tuba Sancı, Ankara Yıldırım Beyazıt University). Fibroblast cells were maintained in the standard medium including DMEM supplemented with 10% heat-inactivated FBS, 2 mM l-glutamine, 100 U/mL penicillin and 100 μg/mL streptomycin. Cultures were incubated at 37 °C in an incubator containing 5% CO_2_ using a BINDER APT.line C150 incubator. The medium was renewed every 2–3 days, and cell morphology was routinely observed under a light microscope. Cells from passages 3 to 5 were used in all experiments.

To establish an *in vitro* hyperglycemic model, L929 fibroblasts were cultured under high-glucose conditions according to previously reported methods.^30^ Cells were maintained in DMEM containing 5.5 mM d-glucose as a normoglycemic control group. To induce hyperglycemic stress, cells were exposed to DMEM with 50 mM d-glucose for 72 h. These experimental conditions were chosen to mimic diabetic-like stress while avoiding excessively cytotoxic effects.^31^

### 2.4 Experimental Design and Treatment Groups

The experimental setup was designed to evaluate the effects of stem cells, bacterial EPSs and their combination therapy under hyperglycemic conditions. Two different EPS preparations were used in this study: EPS1 derived from *L. plantarum* and EPS2 derived from *L. brevis*.

ADMSCs were not directly co-cultured with L929 fibroblasts. Instead, conditioned media were collected from untreated or EPS-treated ADMSCs. ADMSCs were seeded at a density of 1 × 10^5^ cells/well and cultured under the specified conditions. For EPS-preconditioned conditioned media, ADMSCs were exposed to EPS1 or EPS2 at a concentration of 1000 μg/mL during the conditioning period. Following conditioning, the culture supernatants were centrifuged at 300*g* for 5 min to remove cellular debris and designed as EPS1-ADMSC-CM and EPS2-ADMSC-CM, respectively. The collected conditioned media were subsequently applied to hyperglycaemia-induced L929 fibroblasts. Therefore, all subsequent biological analyses, including cell viability, Live/Dead staining, ROS production, apoptosis, and scratch wound healing assays, were performed exclusively on L929 fibroblasts. The experimental groups are summarized in Table 1. All treatments were applied following the establishment of the hyperglycemic model.

**Table 1:** Experimental Groups Used In The Study^a^

group	condition
Control	5.5 mM glucose (normoglycemic)
HG	50 mM glucose (hyperglycemic)
HG + ADMSC-CM	ADMSCs under HG
HG + EPS1	EPS from *L. plantarum* under HG
HG + EPS2	EPS from *L. brevis* under HG
HG + EPS1 + ADMSC-CM	Combination treatment
HG + EPS2 + ADMSC-CM	Combination treatment

a Cells were subsequently maintained under standard culture conditions for the designated experimental periods depending on each assay.

### 2.5 Cell Viability Test

Metabolic activity assay was used to evaluate cellular viability in three sequential experimental stages: (i) determination of non-cytotoxic EPS concentrations in ADMSCs, (ii) evaluation of fibroblast viability under hyperglycemic conditions, and (iii) assessment of combined treatments.

#### 2.5.1 Determination of Non-Cytotoxic EPS Concentrations in ADMSCs

1 × 10^4^ stem cells/well were seeded and expanded in a 96-well plate for 24 h. After adhesion, stem cells were treated with EPS1 or EPS2 at different concentrations (5, 10, 25, 100, 250, 500, and 1000 μg/mL) for 48 h.^32,33^ Following incubation, the Thiazolyl blue tetrazolium bromide (MTT, Biobasic, Canada) reagent (0.5 mg/mL) was introduced to the cells, which were subsequently incubated at 37 °C for 4 h. Formazan crystals were solubilized in dimethyl sulfoxide (DMSO), after which absorbance was recorded at 570 nm using an iMarkTM Microplate Absorbance Reader (Bio-Rad Laboratories). Viability values were normalized to the untreated controls.^30^ Non-cytotoxic concentrations were selected for subsequent experiments.

#### 2.5.2 Cell Viability under Hyperglycemic Conditions

L929 fibroblasts were cultured into 96-well microplates at a seeding density of 1 × 10^4^ cells per well and allowed to adhere for 24 h. Hyperglycemic conditions were induced with 50 mM d-glucose for 72 h. Following model induction, treatment groups defined in Table 1 (including ADMSC-CM, EPS1, EPS2, and their combinations) were applied for an additional 72 h under standard conditions. Metabolic activity was evaluated using the MTT assay following a previously reported method.^30^ Following the period, MTT reagent was performed on the cells for 4 h. Subsequently, formazan crystals were solubilized using DMSO, and absorbance values were recorded at a wavelength of 570 nm (iMarkTM Microplate Absorbance Reader). Viability was presented as a percentage compared with control group. Each experiment was repeated three times.

#### 2.5.3 Live/Dead Cell Staining Assay

L929 fibroblasts were cultured into 24-well microplates at a seeding density of 1 × 10^5^ cells per well and allowed to adhere for 24 h. A hyperglycemic environment was then established by a 72-h treatment with 50 mM d-glucose to the cells. Following model induction, treatment groups defined in Table 1 were applied for an additional 72 h under standard conditions.

After treatment, cell viability was evaluated using a live/dead viability/cytotoxicity kit (Nutri-Culture) according to the manufacturer’s instructions. Cells were incubated in staining medium at 37 °C for 30–45 min under light-protected conditions. Live cells were visualized by calcein-AM fluorescence (excitation/emission: 494/517 nm), while dead cells were detected using propidium iodide (528/617 nm). Nuclei were counterstained with DAPI (4′,6-diamidino-2-phenylindole) for 15 min, and fluorescence imaging was acquired using a Zeiss LSM900 confocal laser scanning microscope equipped with a 20× objective.^30^ All fluorescence images were acquired using identical microscope settings to ensure valid comparisons among treatment groups. The mean fluorescence intensity (MFI) of calcein-AM and propidium iodide (PI) fluorescence was quantified from each field of view to evaluate cell viability and cytotoxicity.

#### 2.5.4 Apoptosis Detection by Acridine Orange/Propidium Iodide Staining

Apoptotic cell death was evaluated using acridine orange/propidium iodide (AO/PI) dual staining. Fibroblasts were seeded and treated under the same experimental conditions as described for the Live/Dead Cell Staining assay, including 24 h adhesion, 72 h hyperglycemic induction, and 72 h treatment period. Treated cells were gently cleansed with phosphate-buffered saline (PBS) and subjected to AO/PI solution for 10–15 min in the dark at room temperature. AO/PI staining solution was prepared according to the manufacturer’s recommendations. Following incubation, cells were immediately visualized using Zeiss/LSM900 confocal laser scanning microscope equipped with a 20× objective. All fluorescence images were acquired using identical microscope settings to ensure valid comparisons among treatment groups. Fluorescence images were analyzed using ImageJ software for quantitative assessment of viable, apoptotic and necrotic cell populations. Viable cells exhibited green fluorescence with normal nuclear morphology, apoptotic cells were identified by condensed or fragmented nuclei with orange fluorescence and necrotic cells displayed red fluorescence because of loss of membrane integrity, according to previously reported morphological criteria.^34^

### 2.6 Wound Closure (Scratch) Assay

Cell migration and wound closure were evaluated using an in vitro scratch assay under hyperglycemic conditions. L929 fibroblasts were seeded into 24-well plates at a density of 1 × 10^5^ cells/well and incubated at 37 °C for 24–48 h until reaching 80% confluence. To mimic a diabetic-like microenvironment, cells were pre-treated with 50 mM d-glucose for 72 h. A linear scratch was manually created using a sterile 200 μL pipette tip, leading to a cell-free gap of approximately 1.5 ± 0.5 mm. Cellular debris was removed by washing with 1× PBS, followed by the addition of fresh culture medium. After scratch formation, the treatment groups described in Table 1 were applied. Cell movement was captured at 0, 24, and 48 h under a phase-contrast microscope. Images were captured, and wound areas were quantified using ImageJ software. The percentage of wound closure was quantified by using the equation^35^

((initialwoundarea−wound⁢areaattimet)/initialwoundarea)×100

### 2.7 Intracellular Reactive Oxygen Species (ROS) Analysis

Intracellular ROS levels were evaluated using the 2′,7′-dichlorodihydrofluorescein diacetate (DCFH-DA) assay according to Liu et al.^36^ L929 fibroblasts were cultured into 24-well microplates at a seeding density of 1 × 10^5^ cells per well for 24 h. To create hyperglycemic environment, treatment with 50 mM d-glucose was applied to cells for a period of 72 h at 37 °C in 5% CO_2_. Following hyperglycemic model induction, treatment groups defined in Table 1 (ADMSC-CM and/or EPS) were applied for 72 h. After treatment, cells were incubated with 10 μM DCFH-DA working solution at 37 °C for 30 min while protected from light. Excess dye was removed by washing with PBS. Fluorescence imaging was performed using Zeiss/LSM900 confocal laser scanning microscope at excitation/emission wavelengths of 485/535 nm. All fluorescence images were acquired using identical microscope settings to ensure valid comparisons among treatment groups. ROS fluorescence was quantified using ImageJ software by measuring the mean fluorescence intensity (MFI) of each field of view. ROS levels were displayed as relative fluorescence intensity compared to the control group. Each experiment was conducted in triplicate.

### 2.8 Statistical Analysis

All statistical evaluations were performed using GraphPad Prism 8.4.3 software (GraphPad Software, San Diego, CA, USA). All data were first controlled for normal distribution. One-way analysis of variance (ANOVA) coupled with Tukey’s post hoc test was conducted to assess differences among multiple groups whereas Student’s *t*-test was used for comparisons between two groups. Data are presented as mean ± SEM. A significance level of *p* < 0.05 was used for all analyses. Unless otherwise stated, experiments were performed using three independent biological replicates, with three technical replicate wells per condition in each experiment where applicable.^26^ For imaging-based assays, multiple fields were analyzed per well and field-level measurements were averaged within each biological replicate before statistical analysis.

## 3 Results

### 3.1 EPS Quantification and Characterization

The EPS production, extraction, purification and characterization steps is provided in Supporting (Figure S1) created for the illustration of the experimental workflow. For the characterization of EPS samples, EPS producing *L. plantarum* and *L. brevis* were activated by seeding from stocks and later prepared for the extraction, purification and characterization steps. Characterization of EPS samples were performed with phenol-sulfuric acid method and FT-IR analysis after acetic acid treatment, ethanol precipitation and lyophilization steps of supernatants of *L. plantarum* and *L. brevis*. The total carbohydrate concentrations of the lyophilized EPS samples were quantified using the phenol-sulfuric acid method. EPS quantification was performed using a glucose standard curve as demonstrated in Supporting (Figure S2). Based on the glucose standard curve, the total EPS amount obtained from *L. plantarum* was 1240 ± 30 mg/L, whereas total EPS amount from *L. brevis* was found to be 750 ± 80 mg/L.

The structural configurations of the purified EPS samples were analyzed using FT-IR spectroscopy. Both samples showed characteristic absorption bands within the 4000-400 cm^–1^ region, consistent with typical polysaccharide structures (Figure 1A,B) and was generally consistent with previously reported findings in the literature. The broad band observed approximately 3300 cm^–1^ was correlated with O–H stretching vibrations, showing strong hydrogen bonding within the EPS matrix. Absorption peaks detected between ∼2920 and 2970 cm^–1^ were associated with C–H stretching vibrations of CH_2_ and CH_3_ groups, indicating the presence of aliphatic structures within the EPS matrix.^37,38^ Furthermore, signals in the region of 1650–1540 cm^–1^ showed C=O stretching and N–H bending vibrations of amide I and amide II groups, suggesting the presence of protein-related components in the EPS structure.^28,39,40^ Bands around ∼1450–1390 cm^–1^ were correlated with C–H bending vibrations. Strong absorption signals observed between 1200 and 1000 cm^–1^ exhibited characteristic features of C–O and C–O–C stretching vibrations, supporting the presence of glycosidic bonds and pyranose ring structures commonly found in microbial polysaccharides.^39,41^ Furthermore, peaks detected below 900 cm^–1^ were associated with α- and β-glycosidic bonds and sugar-related structural features. FT-IR analysis confirmed the polysaccharide nature of the EPS samples and showed similar structural profiles despite differences in production yield.

**Figure 1:**
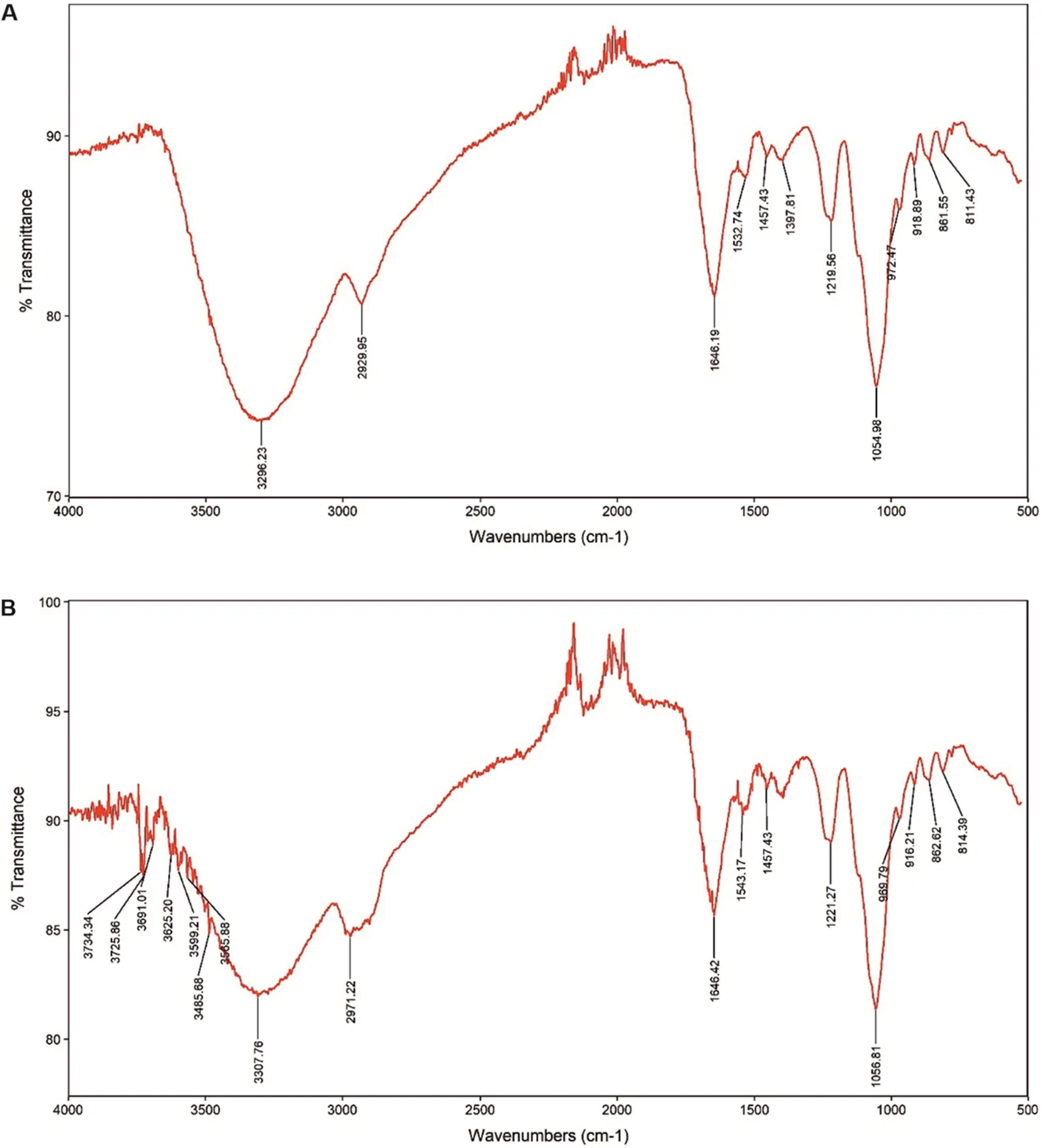
Fourier-transform infrared (FT-IR) spectra of EPS produced by (A) *L. plantarum* and (B) *L. brevis*, confirming the characteristic functional groups related to polysaccharide structures in the purified samples.

### 3.2 Effects of Different Concentrations of EPS1 and EPS2 on ADMSC Viability

The cytocompatibility of purified EPS derived from *L. plantarum* (EPS1) and *L. brevis* (EPS2) was evaluated in ADMSCs using the MTT assay to determine suitable concentrations for subsequent experiments. Cells were treated with increasing EPS concentrations (25, 50, 100, 250, 500, and 1000 μg/mL) for 48 h. Both EPS fractions preserved greater cell viability across all tested concentrations, suggesting that EPS1 and EPS2 were non-cytotoxic in ADMSCs (Figure 2A,B). Cell viability exhibited a slight decrease at low to intermediate EPS concentrations (5–250 μg/mL), followed by an increase at higher concentrations, with the highest viability observed at 1000 μg/mL for both EPS1 and EPS2. Compared with EPS1, EPS2 showed a relatively greater cytocompatibility, particularly at 1000 μg/mL. The concentration of 1000 μg/mL was selected based on the cytocompatibility assessment in ADMSCs and was subsequently used for all treatment groups to ensure experimental consistency.

**Figure 2:**
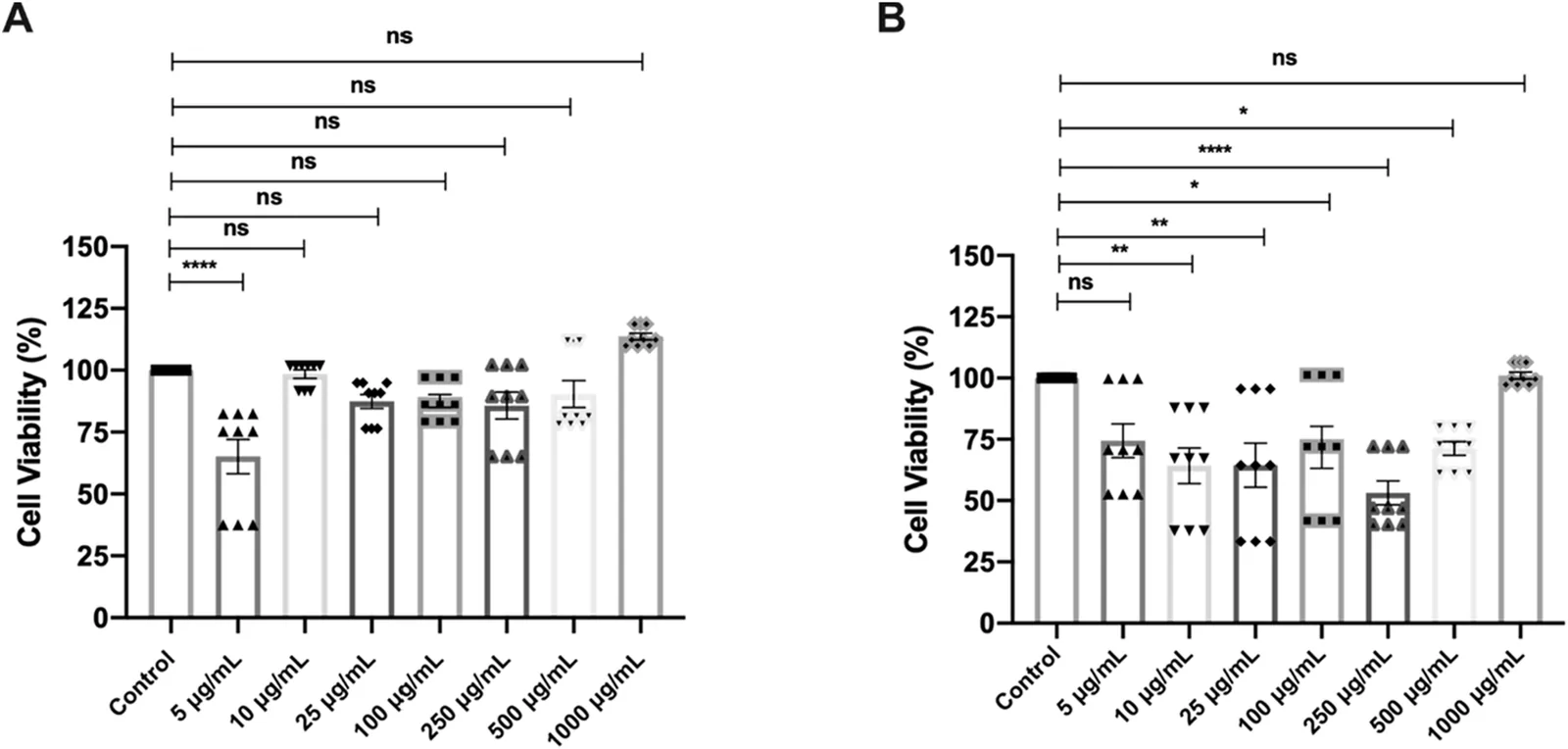
Cytocompatibility and dose–response analysis of EPS obtained from *L. plantarum* (EPS1) and *L. brevis* (EPS2) in ADMSCs assessed by MTT assay. Cells were treated with increasing EPS concentrations (25, 50, 100, 250, 500, and 1000 μg/mL) for 48 h. (A) EPS1 derived from *L. plantarum* preserved high cell viability throughout the tested range, with the highest viability detected at 1000 μg/mL. (B) EPS2 derived from *L. brevis* preserved high cell viability throughout the tested range, with the highest viability detected at 1000 μg/mL. Results are presented as mean ± SEM obtained from three independent experiments conducted in triplicate. Statistical differences among groups were evaluated using one-way ANOVA followed by Tukey–Kramer post hoc analysis. Statistical significance was accepted at *P* < 0.05 (*), *P* < 0.01 (**) and *P* < 0.001 (***).

### 3.3 Combined Application of EPSs and ADMSC-CM Enhances Fibroblast Survival under Hyperglycemic Conditions

To evaluate the protective potential of EPS and ADMSC-CM in a diabetic-like conditions, L929 fibroblasts were subjected to high-glucose medium containing 50 mM d-glucose for 72 h. The non-cytotoxicity of EPSs at 1000 μg/mL, the concentration used for wound closure, was confirmed in L929 fibroblasts (Figure S3). After induction of hyperglycemia, the cells were treated with ADMSC-CM, EPS1, EPS2, or their combinations for an additional 72 h, and cell viability was assessed using MTT assay. High-glucose exposure significantly decreased fibroblast viability when compared with the normoglycemic control group (*P* ≤ 0.01 (**)) (Figure 3A). Treatment with ADMSC-CM alone resulted in an increase in cell viability, while treatment with EPS also improved cell survival under hyperglycemic conditions. EPS2 produced a more remarkable protective effect than EPS1. The combined treatment groups showed stronger cytoprotective effects than the individual treatments. In particular, the combination of EPS2 and ADMSC-CM resulted in the highest viability among all experimental groups to approximately 115% of the levels observed under hyperglycemic-related cellular damage (*P* ≤ 0.0001 (****)) (Figure 3A). Although the combination of EPS1 and ADMSC-CM also significantly increased viability relatived to the untreated hyperglycemic group, its protective effect remained lower than those in the combination of EPS2 and ADMSC-CM.

**Figure 3:**
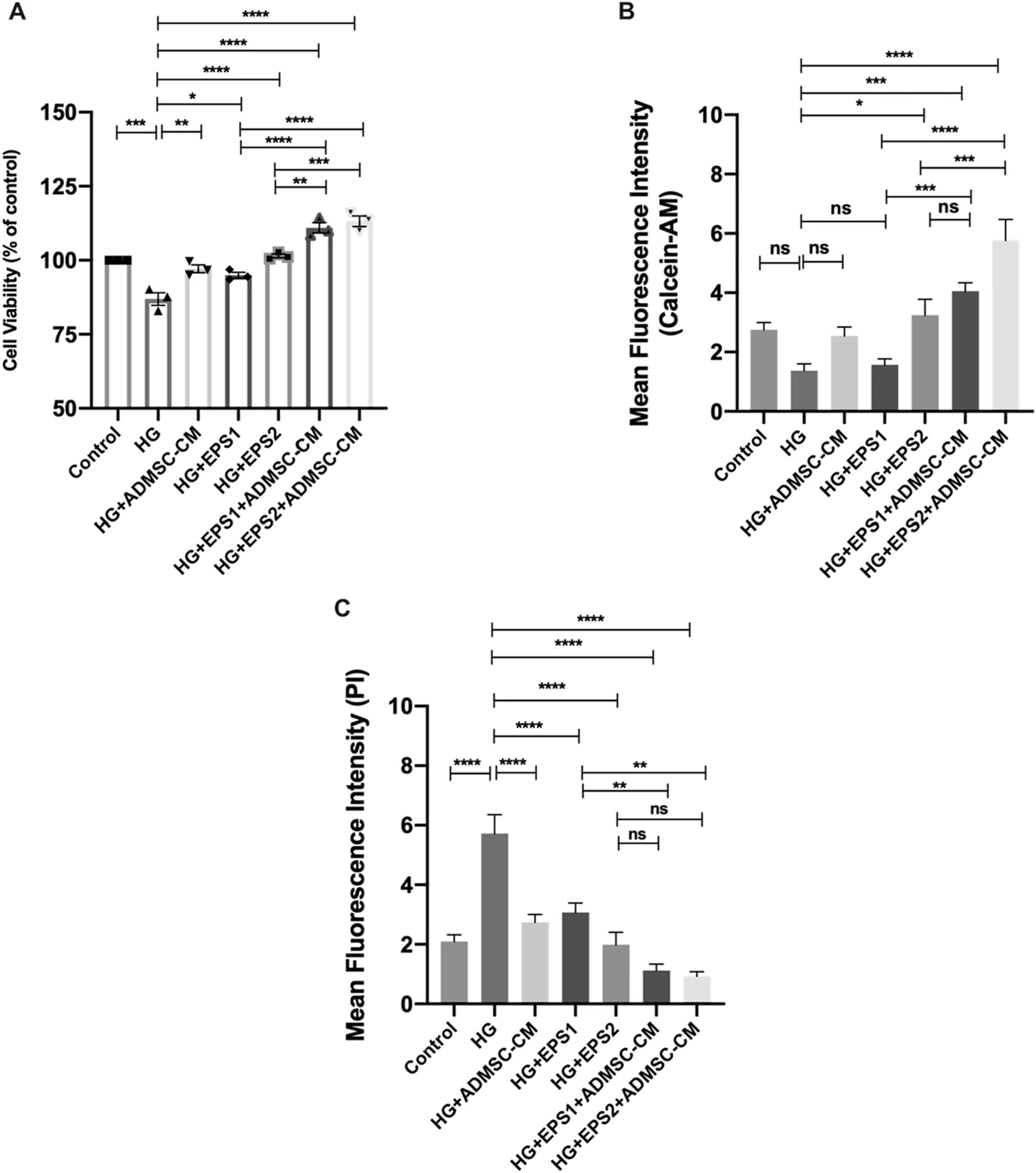
Effects of exopolysaccharides (EPS1 and EPS2) and ADMSC-CM, alone and in combination, on L929 fibroblast viability under hyperglycemic conditions. Cells were cultured under 5.5 mM glucose or 50 mM d-glucose conditions. Following hyperglycemic induction, treatment groups including ADMSC-CM, EPS1, EPS2, and combined EPS-ADMSC-CM formulations were applied. Cell viability was significantly reduced under high glucose conditions, while treatment groups demonstrated partial or significant recovery, with the highest effect observed in combined EPS2 + ADMSC-CM group. (A) The MTT assay was used to evaluate cell viability, and optical density was recorded at 570 nm. (B, C) Quantitative assessment of mean fluorescence intensity of live and dead cell populations based on Calcein AM/PI staining. Results are presented as mean ± SEM from three independent experiments, conducted in triplicate. Statistical differences among groups were evaluated with one-way ANOVA followed by Tukey–Kramer post hoc analysis. Statistical significance was accepted at *P* < 0.05 (*), *P* < 0.01 (**) and *P* < 0.001 (***).

To further confirm the effects of the treatments on cellular integrity and survival, Live/Dead staining was performed using Calcein-AM/PI dual staining and confocal microscopy (Figure 4A–G). Following a 72 h incubation with 50 mM glucose, L929 fibroblast cells were treated with ADMSC-CM, EPS1, EPS2 and their combination for 72 h. Hyperglycemic fibroblasts exhibited reduced viable cell density together with increased PI-positive dead cells and altered cellular morphology compared to the control group (Figure 4A,B). In contrast, treatment groups showed improved cell morphology and increased numbers of viable Calcein-AM-positive cells. Consistent with the MTT findings, the EPS2 + ADMSC-CM group displayed the highest viable cell population and the lowest proportion of dead cells among all hyperglycemic groups (Figures 3B,C and[Fig fig4]G). Quantitative fluorescence analysis further supported the enhanced cytoprotective effect of the combined EPS2 + ADMSC-CM treatment under hyperglycemic conditions (*P* ≤ 0.0001 (****)) (Figure 3B,C).

**Figure 4:**
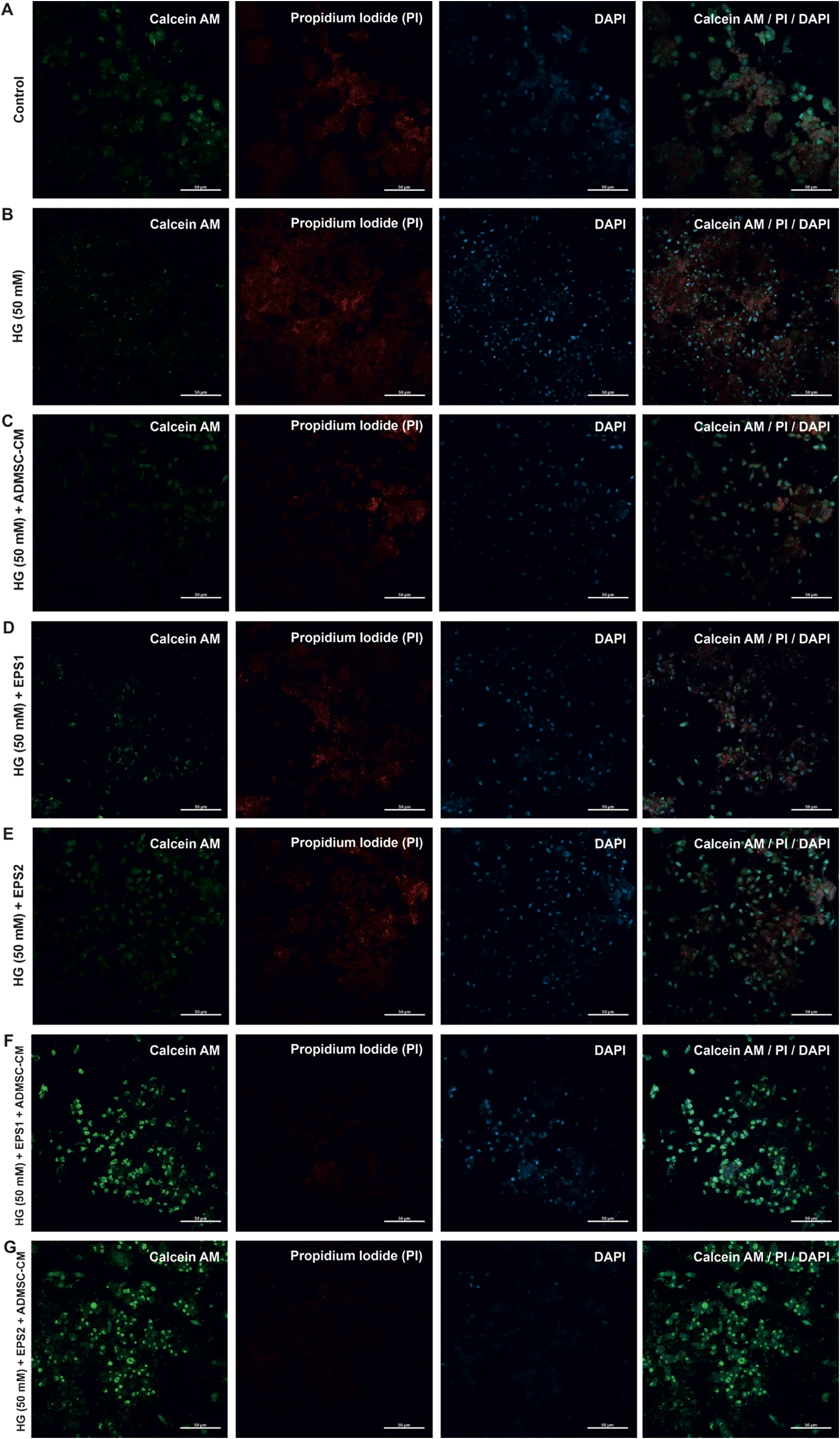
Evaluation of cell viability in L929 fibroblasts under hyperglycemic conditions using live/dead staining and confocal microscopy. Cells were cultured under normal glucose (5.5 mM) or high glucose (50 mM d-glucose) conditions and treated with ADMSC-CM, EPS1, EPS2, and their combinations as described previously. Live cells were visualized with green colour (Calcein-AM), while dead cells were showed with red fluorescence (propidium iodide). Nuclei were counterstained with DAPI (blue fluorescence). (A–G) Representative confocal microscopy images of all experimental groups. The EPS2 + ADMSC-CM group showed the highest viability and the lowest level of cell death under hyperglycemic environment. (A) Control (unstimulated); (B) HG-stimulated fibroblasts (50 mM d-glucose); (C) HG-stimulated fibroblasts with ADMSC-CM; (D) HG-stimulated fibroblasts with EPS1; (E) HG-stimulated fibroblasts with EPS2; (F) HG-stimulated fibroblasts with EPS1 + ADMSC-CM; (G) HG-stimulated fibroblasts with EPS2 + ADMSC-CM. Scale bar = 50 μm.

### 3.4 EPS and ADMSC-CM Co-Treatment Accelerate Scratch Wound Closure under Hyperglycemic Conditions

The effects of EPSs and ADMSC-CM on fibroblast migration and wound closure under diabetic-like conditions were evaluated using an in vitro scratch assay. L929 fibroblasts were first exposed to high-glucose medium (50 mM d-glucose) for 72 h to establish the hyperglycemic wound model. Following scratch formation, treatment groups were applied, and wound closure was monitored at 0, 24, and 48 h. Hyperglycemic conditions markedly impaired fibroblast migration and delayed wound closure compared to the normoglycemic control group (Figure 5). In contrast, treatment with ADMSC-CM or EPS fractions partially improved wound closure and cell migration under high-glucose conditions. Among the individual EPS treatments, EPS2 exhibited a greater promotive effect on fibroblast migration than EPS1. Remarkably, the combined treatment demonstrated greater wound healing compared to treatments applied alone. The EPS2 + ADMSC-CM treatment achieved the highest wound closure rate among all treatment groups after 48 h, indicating enhanced fibroblast migration under hyperglycemic conditions. Furthermore, fibroblasts treated with EPS2 + ADMSC-CM displayed a more organized cellular arrangement and increased migration toward the wound site. Although the EPS1 + ADMSC-CM combination also accelerated wound closure compared with the untreated group (Figure 5A), its effect was less substantial than that observed in the combination treatment with EPS2 and ADMSC-CM. Quantitative analysis performed using ImageJ confirmed that that co-treatment with EPS2 and ADMSC-CM showed a significant increase, reaching 68% wound healing after 48 h, relative to 49% with EPS1 and ADMSC-CM treatment. Notably, these cells (including EPS2, EPS1 + ADMSC-CM and EPS2 + ADMSC-CM) induced a faster rate of healing within the initial 24 h, indicating approximately 20%, 25% and 50% wound healing, respectively (Figure 5B).

**Figure 5:**
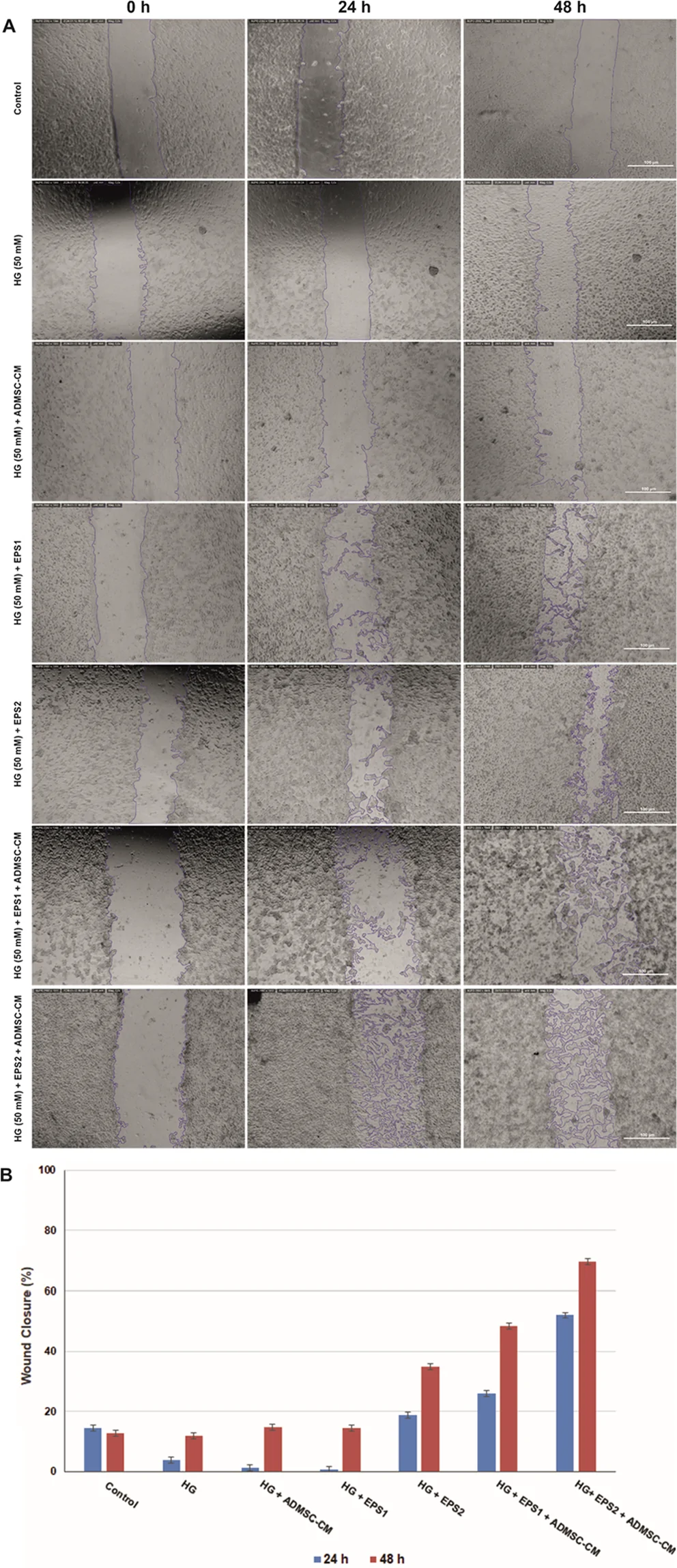
Effects of ADMSC-CM, EPS1, EPS2, and their combinations on fibroblast migration assessed by in vitro scratch wound healing assay in a high-glucose environment. L929 fibroblasts were cultured in a high-glucose (50 mM d-glucose) condition for 72 h, followed by treatment application as described previously. A linear scratch was created, and wound closure was monitored at 0, 24, and 48 h. (A) Representative phase-contrast microscopy images are shown for each time point. Wound areas were recorded using ImageJ software. Scale bar = 100 μm. (B) Quantitative analysis of wound healing was performed. The EPS2 + ADMSC-CM group exhibited the highest wound closure rate compared to all other groups under hyperglycemic conditions. Data are expressed as mean ± SEM from three independent experiments, each with three biological replicates per group.

### 3.5 Combined EPS and ADMSC-CM Treatment under Hyperglycemic Conditions Reduces Intracellular ROS Levels

Intracellular reactive oxygen species (ROS) in hyperglycemic fibroblasts was evaluated using 2′,7′-dichlorodihydrofluorescein diacetate (DCFH-DA) staining and confocal fluorescence imaging. L929 fibroblasts were first exposed to 50 mM d-glucose for 72 h, followed by another 72 h of treatment with ADMSC-CM, EPS1, EPS2, or their combinations. Fluorescence intensity was measured using ImageJ analysis. As shown in Figure 6, fibroblasts cultured under hyperglycemic conditions exhibited markedly increased ROS fluorescence intensity compared to the normoglycemic control group with a fluorescence intensity of 14.95 (**** *P* < 0.0001), indicating enhanced oxidative stress. Treatment with ADMSCs alone moderately reduced intracellular ROS accumulation, while both EPS samples decrease ROS production under high glucose conditions. Among the single treatment groups, they showed similar antioxidant effect (Figure 6A,B). Notably, the combined treatment groups showed a greater reduction in ROS levels compared to individual treatments. The EPS2 + ADMSC-CM group exhibited the lowest fluorescence intensity among all hyperglycemic groups, and ROS levels decreased to values approaching those of the control group (Figure 6B). Pretreatment with 1 mg/mL *L. plantarum* significantly reduced ROS levels, with a fluorescence intensity of 5.91 (**** *P* < 0.0001). The fluorescence intensity of ROS was approximately 9-fold and 4.15-fold lower in EPS2+ADMSC-CM and EPS1+ADMSC-CM groups than the hyperglycemic group (**** *P* < 0.0001) (Figure 6B).

**Figure 6:**
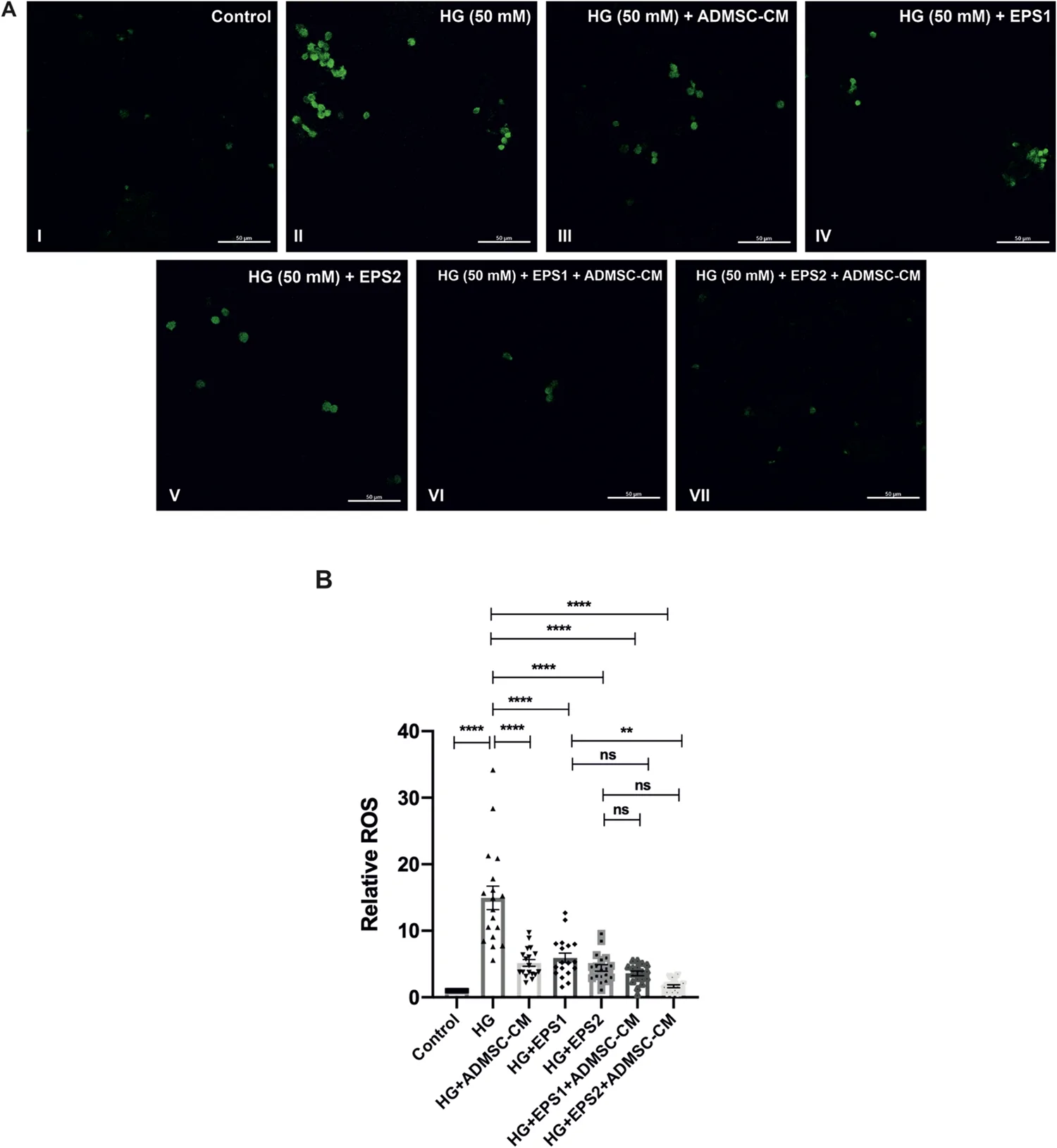
Intracellular reactive oxygen species (ROS) levels in L929 fibroblasts under hyperglycemic conditions following treatment with ADMSC-CM, EPS1, EPS2, and their combinations. Cells were exposed to high glucose for 72 h and then treated as previously described. Intracellular ROS production was measured using a DCFH-DA fluorescence probe. (A) Representative confocal microscopy images showing ROS fluorescence intensity between experimental groups. Scale bar = 50 μm. (B) ROS fluorescence intensity normalized to control group. The EPS2 + ADMSC-CM treatment group significantly reduced ROS levels compared to all other groups. Data are expressed as mean ± SEM from three independent experiments, each performed in triplicate biological replicates. Statistical differences between groups were evaluated using one-way ANOVA followed by Tukey-Kramer post hoc analysis. Statistical significance was accepted at *P* < 0.0001 (****).

Confocal microscopy images also support these findings, showing intense DCF fluorescence in untreated hyperglycemic fibroblasts, while significantly weaker fluorescent signals and improved cellular morphology were observed in the combined treatment groups (Figure 6A). Overall, these results demonstrate that treatment with EPS2 and ADMSC-CM effectively suppresses oxidative stress.

### 3.6 The Combined Administration of EPS and ADMSC-CM Suppresses Apoptotic Cell Death under Hyperglycemic Conditions

Apoptotic cell death in hyperglycemic fibroblasts was evaluated using AO/PI staining followed by confocal microscopic analysis. L929 fibroblasts were subjected to high glucose conditions for 72 h and then treated with ADMSC-CM, EPS1, EPS2, or their combinations for another 72 h. Percentage and mean fluorescence intensity of apoptotic/dead cells were measured using ImageJ software.

As shown in Figure 7B, fibroblasts exposed to hyperglycemic conditions showed an increased apoptotic and dead cell population compared to the control group. Untreated hyperglycemic cells demonstrated intense red fluorescence along with altered cellular morphology, indicating increased membrane damage. Treatment with ADMSC-CM or EPS samples alone partially reduced apoptotic cell accumulation under high glucose conditions (Figure 7C–E). Among individual EPS treatments, EPS2 showed a stronger anti-apoptotic effect than EPS1. Spesifically, combination therapy showed a more pronounced reduction in apoptotic cell populations compared to single therapy groups. The EPS2 + ADMSC-CM group exhibited the lowest percentage of apoptotic cells among all hyperglycemic groups, with predominantly viable cells, preserved cellular morphology (Figure 7G). While the EPS1 + ADMSC-CM combination also reduced apoptotic cell levels compared to hyperglycemic fibroblasts, its protective effect was less than that observed in the EPS2 + ADMSC-CM group (Figure 7F,G). Quantitative analysis confirmed that treatment with EPS2 and ADMSCs in combination significantly suppresses hyperglycemia-induced apoptotic cell death (Figure 8A,B).

**Figure 7:**
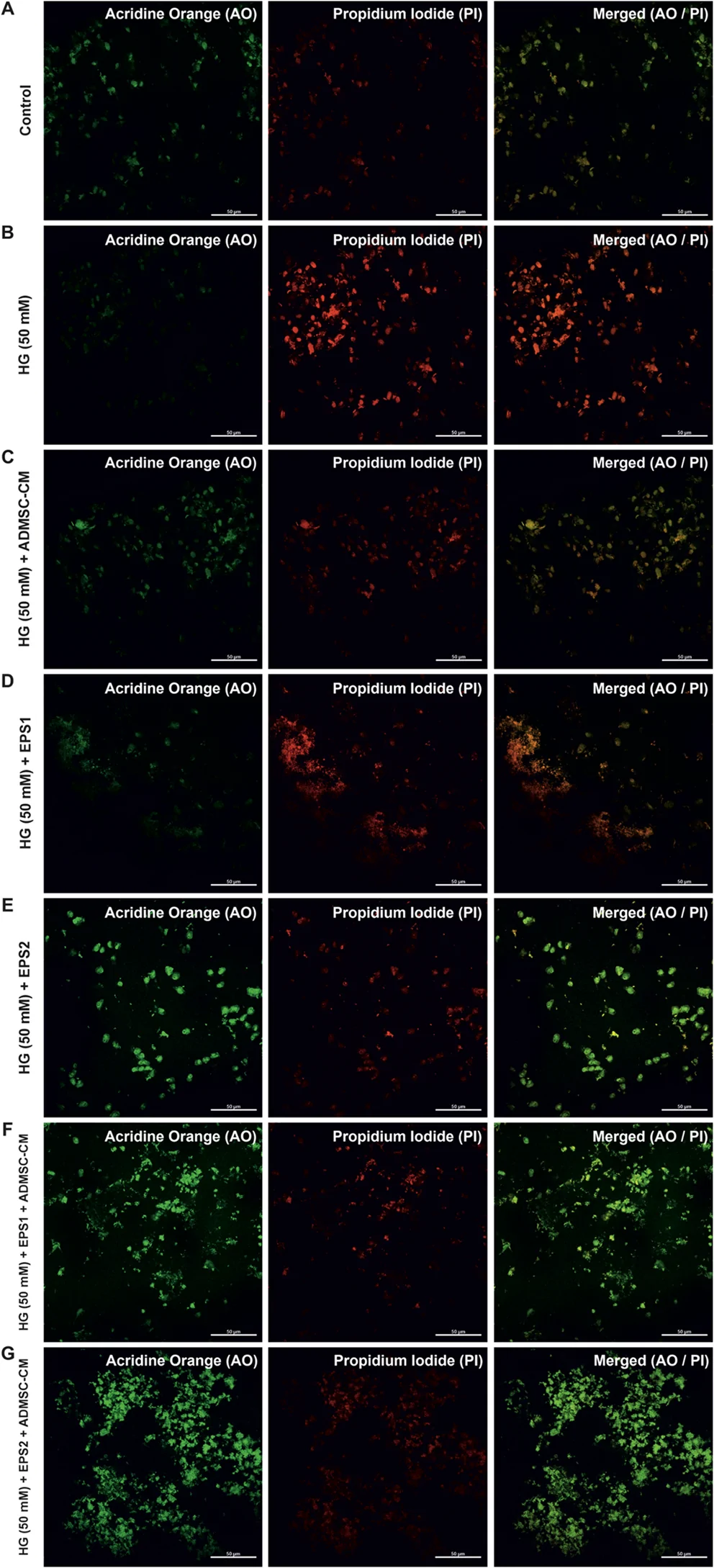
Evaluation of apoptotic/dead cells under hyperglycemic conditions following treatment with ADMSC-CM, EPS1, EPS2, and their combinations. Cells were cultured under high glucose conditions and treated as previously described. Apoptotic and dead cells were detected using acridine orange/propidium iodide (AO/PI) staining. (A–E) Representative confocal microscopy images showing live (green), early apoptotic (yellow), and dead (red) cells with nuclear counterstaining. Scale bar = 50 μm.

**Figure 8:**
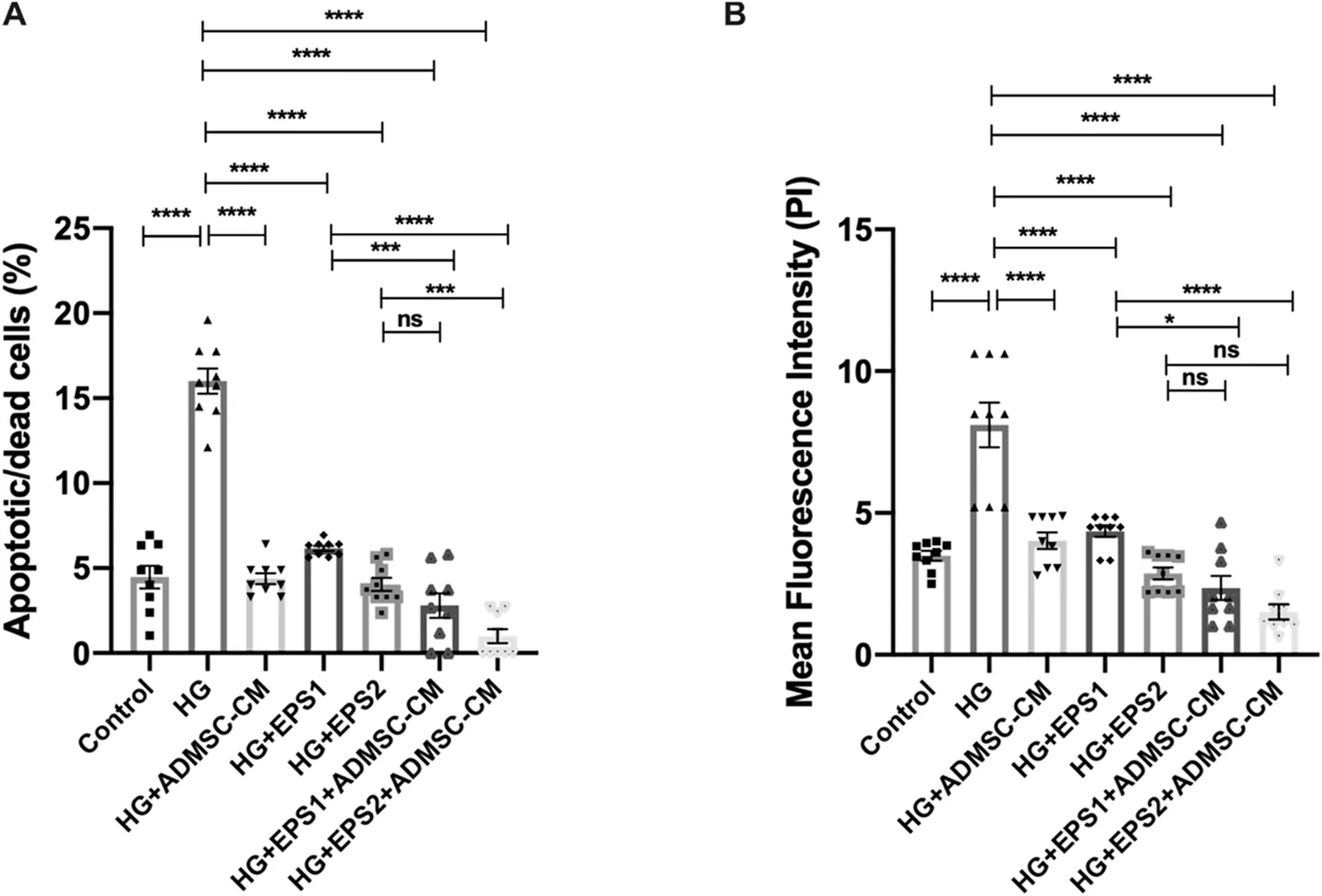
Quantitative analysis of percentage of apoptotic/dead cells among experimental groups. (A) The percentage of apoptotic/dead cells under hyperglycemic conditions. The EPS2 + ADMSC-CM group exhibited the lowest apoptotic/dead cell ratio compared to another groups. (B) Quantitative analysis of mean fluorescence intensity of PI under hyperglycemic conditions. Data are expressed as mean ± SEM from three independent experiments, each performed in triplicate biological replicates. Statistical differences between groups were evaluated using one-way ANOVA with subsequent Tukey-Kramer post hoc analysis. Statistical significance was accepted at *P* < 0.05 (*), *P* < 0.01 (**), *P* < 0.001 (***) and *P* < 0.0001 (****).

## 4 Discussion

Diabetic wounds remain a significant clinical challenge, resulted in a persistent hyperglycemic microenvironment, chronic inflammation, impaired angiogenesis and excessive oxidative stress, all of which hinder normal tissue repair.^2^ Wound healing is a tightly regulated physiological process including inflammatory, proliferative, and remodelling phases. However, in diabetic conditions, persistent hyperglycemia impairs vascular integrity, limits oxygen and nutrient delivery to injured tissues, and reduces fibroblast proliferation and migration, ultimately delaying wound closure and tissue regeneration.^42,43^ In recent years, mesenchymal stem cells (MSCs) have gained increasing interest in regenerative medicine due to their immunomodulatory, anti-inflammatory and pro-regenerative functions. However, the diabetic wound environment restricts the survival and therapeutic potential of MSCs after transplantation. These limitations have increased interest in supportive biomaterials that can enhance cellular function and improve tissue repair.

Among these biological agents, probiotic-derived exopolysaccharides (EPSs) have emerged as promising bioactive compounds due to their antioxidant, anti-inflammatory, and tissue regenerative properties.^44^ Previous studies have demonstrated that EPSs derived from lactic acid bacteria promote collagen deposition, stimulate fibroblast activity, and accelerate wound shrinkage, while also exhibiting anti-elastase and anti-collagenase effects.^20,44^ EPS obtained from *L. plantarum* has also been reported to increase fibroblast and keratinocyte proliferation and migration without producing cytotoxic effects.^23^ Similarly, EPS produced from *L. brevis* has been shown to improve granulation tissue formation, increase fibroblast activity, and enhance wound closure in experimental wound models.^21^ However, the potential effects of combining probiotic EPS with ADMSCs in diabetic wound conditions have not yet been comprehensively investigated.

In this experimental study, the ameliorative effect of EPS purified from *L. plantarum* and *L. brevis* was evaluated in conjunction with ADMSCs. Cytocompatibility analyses showed that both EPSs maintained ADMSC viability at the tested concentrations, with the highest viability observed at 1000 μg/mL (Figure 2A,B). In particular, EPS2 from *L. brevis* showed a stronger proliferative effect than EPS1. Variations in EPS biological activity have been associated with differences in strain origin, molecular architecture, monosaccharide composition, degree of branching and molecular weight in previous studies.^45,46^ Although comprehensive structural and purity characterizations including monosaccharide composition, molecular weight distribution, and endotoxin determination were not included in the present study, it is reasonable to speculate, based on previous reports, that structural differences between the EPS fractions may contribute to their distinct effects on stem cell compatibility. However, this hypothesis requires further investigation through detailed structural analyses. Consistent with previous reports,^31^ high glucose exposure significantly reduced fibroblast viability and increased cellular damage in this study (Figure 3A–C). Live/Dead staining confirmed the increased cell death with marked changes in fibroblast morphology under hyperglycemic conditions. Although treatment with ADMSC-CM or purified EPS alone improved cell survival, combination therapy produced a more significant protective response (Figures 4C–G). Among all experimental groups, EPS2 combined with ADMSC-CM resulted in the greatest improvement in fibroblast viability and cellular morphology, demonstrating the regenerative potential of a possible interaction mediated by stem cells and EPS. Fibroblast migration plays a critical role in granulation tissue formation and wound closure during the proliferative phase of healing.^7,47,48^ In diabetic wounds, prolonged oxidative stress and chronic inflammation disrupt fibroblast motility and delay re-epithelialization.^1^ Consistent with these pathological features, scratch assay demonstrated that hyperglycemic conditions significantly reduced wound closure capacity compared to normoglycemic controls. While individual treatment with EPS fractions or ADMSC-CM slightly improved fibroblast migration, co-treatment with EPS2 and ADMSC-CM resulted in the highest wound closure rate at 48 h (Figure 5B). Microscopic observations also revealed enhanced cellular organization and more directed migration toward the wound area in the combined treatment groups (Figure 5A). Collectively, these findings indicate that EPS2 may help establish a regenerative microenvironment that supports ADMSC-mediated repair under diabetic conditions.

Oxidative stress plays a central role in the progression of diabetic wounds by inducing mitochondrial dysfunction, DNA damage and apoptosis.^1,16^ Excessive intracellular ROS accumulation disrupts normal fibroblast activity and contributes to the development of chronic non-healing wounds. In the present study, untreated hyperglycemic fibroblasts exhibited markedly elevated intracellular ROS levels, consistent with the oxidative burden in a high-glucose environment (as shown in Figure 6A). Although both EPS and ADMSC-CM treatments reduced ROS production, the combination of EPS2 and ADMSC-CM produced the most significant antioxidant response, with an approximately 9-fold reduction in ROS fluorescence intensity compared to the hyperglycemic group (Figure 6B). The antioxidant properties of LAB-derived EPSs have been associated with their reducing potential, radical scavenging capability and hydroxyl group content.^12,44^ Accordingly, the significant reduction in intracellular ROS observed in the combined treatment group may contribute to enhancing wound repair potential. In addition to oxidative stress, apoptotic cell death is another critical factor limiting tissue regeneration in diabetic wounds. AO/PI staining demonstrated that hyperglycemic conditions significantly increased apoptotic and dead cell populations, while treatment with ADMSC-CM and the purified EPS reduced this effect (Figure 7B–E). The strongest anti-apoptotic response was observed in the EPS2 + ADMSC-CM group, exhibiting significantly reduced PI-positive staining and preserved cellular morphology (Figures 7G and 8B). These findings suggest that combining ADMSC-CM with probiotic-derived EPSs may provide regenerative support in diabetic wound. Consistent with our results, MSC-based therapies have previously been reported to reduce apoptosis and promote tissue regeneration via paracrine signalling mechanisms.^5,6^

The findings of this study demonstrate that combining probiotic-derived EPSs with ADMSC-CM enhances fibroblast viability, suppresses oxidative stress and apoptosis, and accelerates wound healing under hyperglycemic conditions. Among the tested EPS fractions, EPS2 from *L. brevis* consistently showed superior biological activity, particularly when combined with ADMSC-CM. To our knowledge, this study is one of the first to evaluate the combined regenerative potential of purified LAB-derived EPS and ADMSC-CM in a diabetic fibroblast model. However, several limitations should also be considered. Although the current study provides important preliminary findings, it was performed using an *in vitro* fibroblast model and therefore does not fully reflect the complex cellular and immunological dynamics of diabetic wounds healing *in vivo*. Furthermore, extracellular matrix remodeling and myofibroblast differentiation were not investigated in the present study. Further studies should include regeneration-related markers, such as collagen deposition, α-smooth muscle actin (α-SMA) expression, and TGF-β1/SMAD signalling, to further elucidate the molecular mechanisms underlying the observed wound healing effects. Additional studies using primary human cells and diabetic animal models will also be important to validate the translational potential of this combined therapeutic strategy. This study is aligned with the United Nations Sustainable Development Goal 3 (SDG 3: Good Health and Well-being) by supporting research on novel approaches for diabetic wound management.

## 5 Conclusion

This study demonstrated that probiotic-derived EPS, specifically purified from *L. brevis*, and ADMSC-CM enhanced their therapeutic effects under hyperglycemic conditions. Combined treatment with EPSs and ADMSC-CM improved fibroblast viability, supported wound healing and promoted cell migration in a diabetic wound model by reducing intracellular ROS accumulation and suppressing cell death. The combination of EPS derived from *L. brevis* with ADMSC-CM consistently showed the most potent protective and regenerative effects. These findings suggest that combining probiotic-derived biomolecules with stem cell-based approaches may be a promising strategy for diabetic wound healing. The observed effects indicate that EPS can support the diabetic wound microenvironment by enhancing cellular survival and reducing oxidative stress–related damage. However, further studies using primary human cells and *in vivo* diabetic wound models are needed to validate the underlying molecular mechanisms and evaluate the translational potential of this combined therapeutic approach.

## Supplementary Material



## Notes

Schematic illustration of the experimental design for EPS production and characterization created with BioRender.com (Figure S1); glucose standard curve for the quantification of EPS samples (Figure S2); cytotoxicity analysis of EPSs at the concentrations used for wound closure in L929 fibroblasts (Figure S3) (PDF)
