# Supplementary material for: Probiotic-Derived Exopolysaccharides Enhance Mesenchymal Stem Cell-Mediated Fibroblast Repair under Hyperglycemic Conditions
Source: ACS Omega. 2026 Sep 29;11(40):61586–99. doi: 10.1021/acsomega.6c07182 (PMC13670295; doi:10.1021/acsomega.6c07182)

Supplementary Material

Probiotic-Derived Exopolysaccharides Enhance Mesenchymal Stem Cell-Mediated Fibroblast Repair Under Hyperglycemic Conditions

Mesude Bicer [1,*], Mert Akdur [1,#], Esra Battal [1,#], Fatma Sener [2], Irem Yilmaz [2], Özkan Fidan [1]

[1] Department of Bioengineering, Faculty of Life and Natural Sciences, Abdullah Gul University, Kayseri, Turkiye, 38080
[2] Department of Bioengineering, Graduate School of Abdullah Gul University, Abdullah Gul University, Kayseri, Turkiye, 38080

[#]These authors contribute equally.
[*] Correspondence: mesude.bicer@agu.edu.tr

Figure S1 Schematic illustration of the experimental design for EPS production and characterization. Created with BioRender.com.

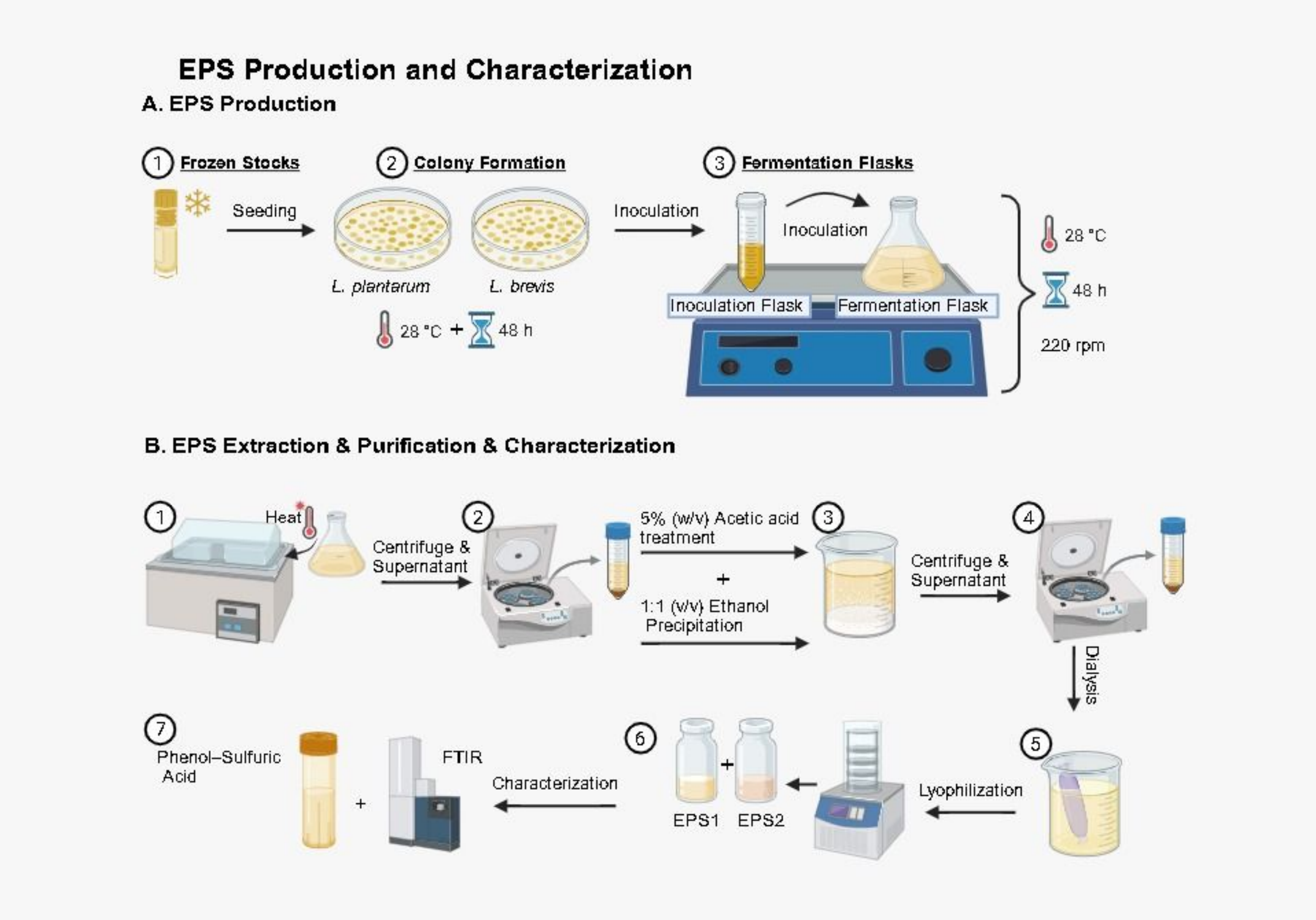

Figure S2 Glucose standard curve for the quantification of EPS samples

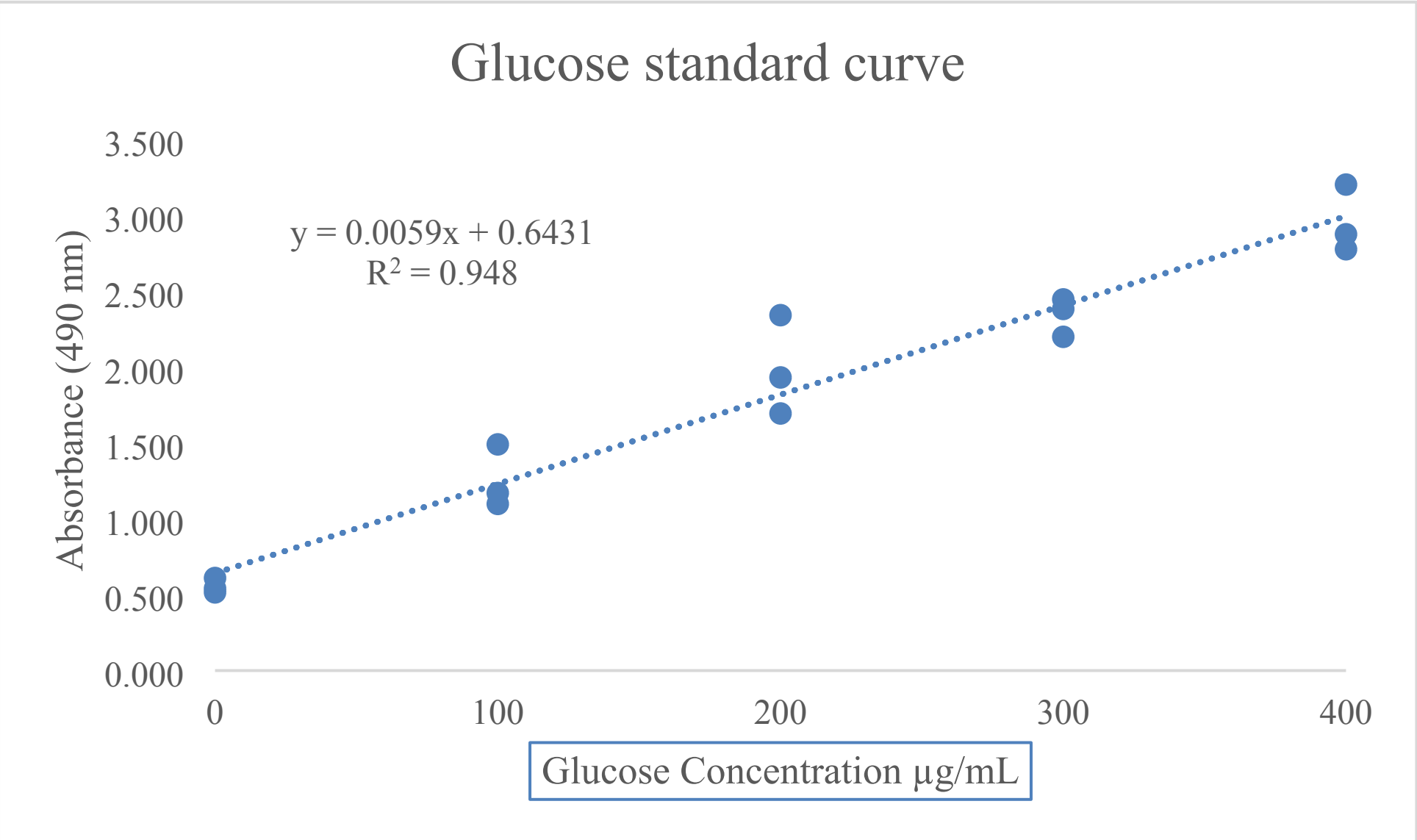

Figure S3 Cytotoxicity analysis of EPSs at the concentrations used for wound closure in L929 fibroblasts

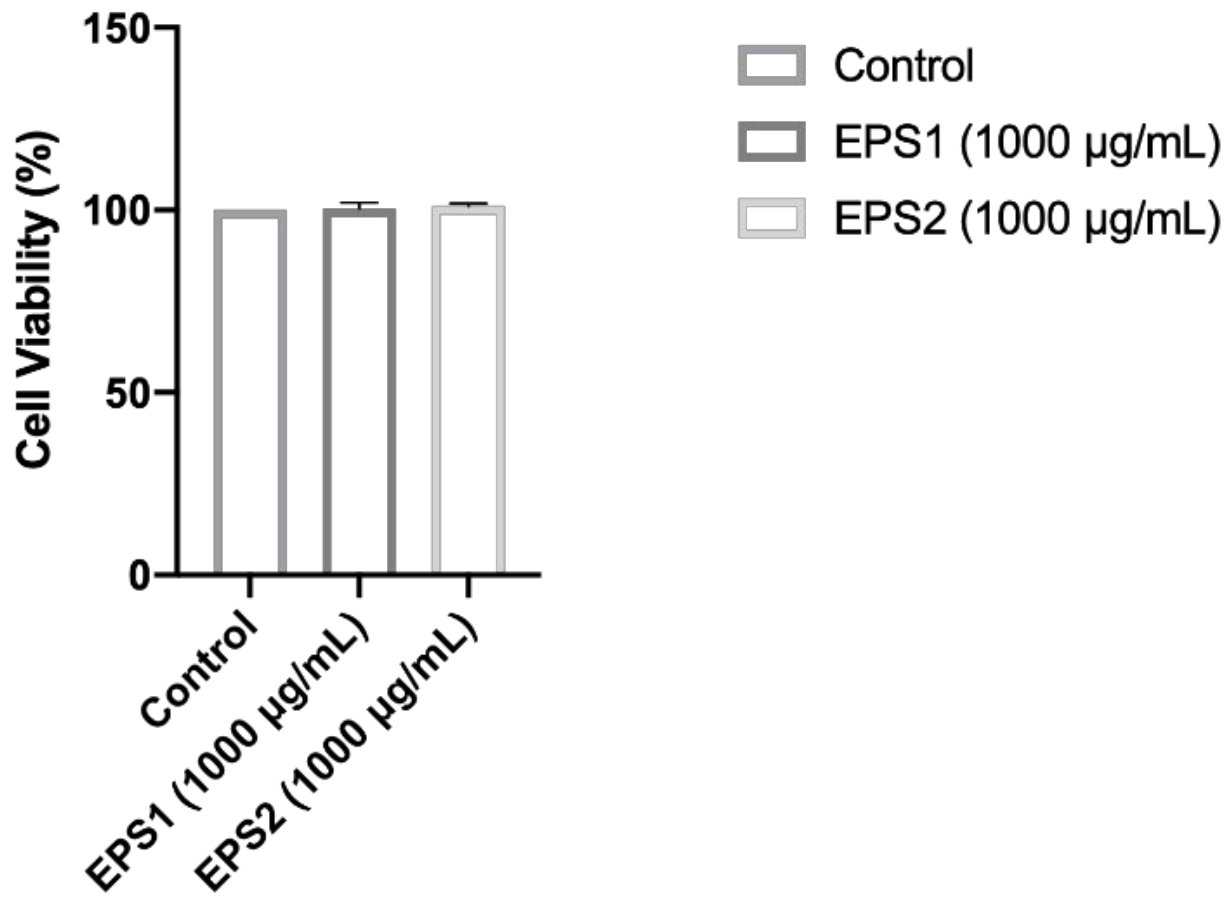

Supplement: Supplementary file 1 [file ao6c07182_si_001.pdf]
